# Inhaled Corticosteroid use and the Risk of Pneumonia and COPD Exacerbations in the UPLIFT Study

**DOI:** 10.1007/s00408-017-9990-8

**Published:** 2017-03-03

**Authors:** Jaymin B. Morjaria, Alan Rigby, Alyn H. Morice

**Affiliations:** 1 Centre for Cardiovascular & Metabolic Research, Hull York Medical School, University of Hull, Castle Hill Hospital, Castle Road, Cottingham, HU6 5JQ UK; 2 Department of Respiratory Medicine, Royal Brompton & Harefield NHS Trust, Harefield Hospital, Hill End road, Harefield, UB9 6JH UK

**Keywords:** Fluticasone, Inhaled corticosteroids, Tiotropium, UPLIFT, Pneumonia, COPD

## Abstract

**Rationale:**

Unlike many other COPD studies, the 4-year UPLIFT trial permitted inhaled corticosteroid (ICS) use during run-in and treatment phases. This provided the opportunity to prospectively observe the continuing effects of ICS on respiratory events in closely observed COPD population.

**Objectives:**

We aimed to determine rate and number of episodes of pneumonia and exacerbations of COPD in patients entering the study on no ICS, fluticasone proprionate (FP), and other ICS.

**Methods:**

The UPLIFT dataset was examined retrospectively, and patients were divided into three groups based on their medications at entry: no ICS, FP and other ICS. Poisson regression was used to compare the frequency of respiratory adverse events.

**Measurements and Main Results:**

At entry, the groups were well matched apart from a higher FEV1% predicted (38 vs. 41%; ICS vs. no ICS, respectively) and prevalence of current smoking (26 vs. 36%; ICS vs. no ICS, respectively). Incidence rates of pneumonia were significantly higher in patients taking ICS compared to no ICS (0.068 vs. 0.056 respectively; *p* = 0.012). When the FP group was compared to the other ICS, the event rate was even higher (0.077 vs. 0.058, respectively; *p* < 0.001). COPD exacerbations were more frequent in patients taking ICS, with significantly greater rate in the FP group compared to that seen with other ICS (0.93 vs. 0.84 respectively; *p* = 0.013).

**Conclusions:**

ICS use was associated an increase in respiratory adverse event rates, but whether this was due to more severe illness at entry is unknown. In subgroup analysis, the excess of morbidity in the ICS group appeared to be mainly associated with those receiving FP at randomisation.

**Electronic supplementary material:**

The online version of this article (doi:10.1007/s00408-017-9990-8) contains supplementary material, which is available to authorized users.

## Introduction

Chronic obstructive pulmonary disease (COPD) is characterised by poor and worsening lung function, and significant patient and societal burdens [1]. Prevention and treatment of exacerbations have been identified by the Global Initiative for Chronic Obstructive Lung Disease (GOLD) as a priority since they are associated with lung function decline. The recommendation of inhaled corticosteroid/long-acting β_2_ agonist combinations (ICS/LABA) is based on evidence of a reduction in exacerbations in studies such as TORCH [2]. Re-analyses of studies such as the TORCH have cast doubt on whether the corticosteroid component of these combinations, is efficacious [3]. However, ICS and ICS/LABA combinations are commonly prescribed inappropriately early in the course of the disease where bronchodilator therapies (short acting bronchodilators, long-acting muscarinic antagonists (LAMAs), LABAs) may have been more suitable [4, 5]. The recent results of the FLAME study call this strategy into further question [6]. Not only has the efficacy of ICS in COPD, especially mild-to-moderate disease, remained open to question [4], but their use especially at the high doses recommended in COPD are now associated with significant adverse events such as pneumonia, cataracts, glaucoma, accelerated bone turnover and diabetes [7]. These are acknowledged in current management strategies and in guidelines to a varying extent, although there is no clear guidance on how the increased risk should inform decision-making by physicians [1, 8].

More recently, with respect to pneumonia, there has been debate as to whether all ICS carry a similar risk profile and whether these are dose-related. The majority of studies demonstrating increased pneumonia risk were performed with fluticasone propionate (FP) doses (500–1000 µg/day), including the 3-year TORCH and 2-year INSPIRE studies [2, 9–13]. Meta-analyses of randomised studies have reported an increased pneumonia risk of up to 70% with ICS use [14, 15]. However no or lower risk of pneumonia have been noted with budesonide compared to FP [16, 17]. Similar observations have been made from retrospective database analyses, and observational matched primary care medical records review [18, 19]. Of the studies of budesonide and beclomethasone in COPD, only two have demonstrated a significant increase in reported pneumonia [20, 21].

Despite the growing evidence that FP-based therapies have higher rates of pneumonia criticisms have been raised including the lack of clear clinical information from databases, poor patient-control matching, the retrospective nature of all analyses, and the mix of protocol designs comparing ICS/LABA versus their monotherapies and/or placebo. Indeed, the view of the European Medicines Agency, which adopted the Committee for Medicinal Products for Human Use, was that there was no difference in interclass respiratory adverse events between ICS [22]. In 2008, the then largest randomised controlled study of COPD patients, the UPLIFT study, was published [23]. An unusual feature of this 4-year study was that patients were maintained on existing non-cholinergic inhaled therapies whilst being randomised to tiotropium or matched placebo. We hypothesised that a comparison of patients taking ICS at entry to the study would allow comparison of the rates of adverse respiratory events in patients taking FP compared to those on other ICS.

## Methods

### Clinical Study Design and Subjects

The study designs, recruitment criteria and procedures of the UPLIFT studies have been previously reported (UPLIFT clinical Trial registration: NCT00144339) [23]. Briefly, this was a multi-centre 4-year double-blind, parallel-group study in COPD patients with moderate-to-severe airflow limitation randomised to placebo or tiotropium 18 µg once daily. The two co-primary endpoints were the annual rate of decline in pre-bronchodilation forced expiratory volume in 1 s (FEV1) and the post-bronchodilator FEV1 after 30 days of randomisation to the completion of the study. Importantly, the subjects were permitted guideline-recommended therapies other than the study drug (tiotropium or placebo) and other than any alternative anti-cholinergic therapies. All subjects were ≥40 years of age, had a diagnosis of COPD, smoked for ≥10 pack years, were not on long-term oxygen therapy and had not had an exacerbation of COPD or respiratory infection within the last 4 weeks of screening.

Exacerbations in the study were characterised as an increase in new onset of at least two or more symptoms of cough, sputum, sputum purulence, wheezing or dyspnoea for ≥3 days requiring additional treatment with antibiotics and/or systemic corticosteroids. Pneumonia was defined based on the investigator’s assessment of a respiratory adverse event.

### Statistical Methods

Because of the greater certainty in obtaining baseline prescribing data, it was decided that the analyses would be undertaken on an intention-to-treat basis. Baseline continuous data are summarised by the median (25th /75th centiles); categorical data by *n* (%). Three different categories were analysed:

1. ICS (*n* = 3700) vs no ICS (*n* = 2292);
2. Within ICS, FP (*n* = 1981) versus other ICS (or non-FP) (*n* = 1719);
3. Stratification of the latter three groups by presence/absence of tiotropium, thus giving rise to six groups.

The relationship between the treatment groups (*n* = 2, 3 or 6) and the frequency of events (pneumonia and COPD exacerbations) was analysed by Poisson regression. An assumption of Poisson data is that the mean number of events is equal to the variance. The question of overdispersion of Poisson data is addressed in the supplementary section of this study. Time-to-first event was plotted by Kaplan–Meier curves [24]; the Log-rank test was used to compare treatment groups. Statistical analysis of COPD exacerbations followed those for pneumonia except for incidence rates which are presented per person-year because of the high frequency of exacerbations. Cox proportional hazards regression was used for COPD exacerbations from which hazard ratios (HRs) and 95% CIs estimated. The data were rounded up to the nearest whole number. An arbitrary level of 5% statistical significance (two-tailed) was assumed. The Stata statistical computer package was used to analyse the data.

## Results

### Demographics and Other Baseline Characteristics

This is an analysis of a study which was not stratified at entry so some baseline between-group differences are to be expected. We anticipated that the ICS group would have greater morbidity than the non-ICS group. Baseline variables are given in Table 1. The average age was similar across groups with the proportion of men varying between 72 and 76%, and the majority of patients were Caucasian. There were proportionately more current smokers in the no ICS group (36%) compared to the ICS group (26%). FEV1% predicted was 3% lower on average for patients on any ICS when compared to no ICS (38 vs. 41%; ICS vs. no ICS, respectively). Thus, there were proportionately more severe patients and fewer smokers in the ICS group compared to those with no ICS.

**Table 1:** Baseline demographics

Variable	Fluticasone		Other ICS		No ICS	
	Placebo	Tiotropium	Placebo	Tiotropium	Placebo	Tiotropium
	(*n* = 987)	(*n* = 994)	(*n* = 873)	(*n* = 846)	(*n* = 1146)	(*n* = 1146)
Age (years)	65 (59, 71)	66 (59, 71)	65 (59, 71)	65 (59, 71)	65 (58, 71)	64 (58, 70)
Age ≥70	297 (30%)	315 (32%)	280 (33%)	269 (31%)	352 (31%)	311 (27%)
Sex (men)	716 (73%)	714 (72%)	653 (77%)	652 (75%)	853 (74%)	885 (77%)
Race						
White	895 (91%)	912 (92%)	804 (92%)	769 (91%)	998 (87%)	1010 (88%)
Black	19 (2%)	13 (1%)	4 (<1%)	50 (10%)	96 (8%)	99 (10%)
Asian	48 (5%)	43 (4%)	41 (5%)	21 (3%)	22 (2%)	18 (2%)
Unknown	3 (<1%)	26(3%)	24 (3%)	21 (3%)	22 (2%)	18 (2%)
Smoker current	242 (25%)	247 (25%)	234 (28%)	214 (25%)	422 (37%)	413 (36%)
FEV1 (L)	1.02 (0.76, 1.33)	0.99 (0.76, 1.29)	1.01 (0.75, 1.35)	1.05 (0.79, 1.34)	1.1 (0.83, 1.39)	1.1 (0.83, 1.43)
FEV1PostBD	1.24 (0.94, 1.58)	1.22 (0.96, 1.57)	1.26 (0.94, 1.61)	1.3 (1.159)	1.36 (1.01, 1.65)	1.35 (1.05, 1.7)
FEV1% Pred	38 (29, 47)	38 (29, 47)	38 (29, 47)	39 (30, 48)	41 (32, 50)	40 (32, 51)
FEV1%predBD	36 (36, 56)	47 (37, 57)	47 (26, 57)	48 (37, 57)	50 (41, 59)	50 (40, 60)
FVC (L)	2.52 (2.02, 3.13)	2.5 (1.94, 3.07)	2.51 (2, 3.12)	2.59 (2.06, 3.15)	2.6 (2.03, 3.19)	2.62 (2.07, 3.15)
FVC% Pred	74 (62, 86)	73 (61, 86)	73 (60, 85)	74 (62, 86)	76 (64, 88)	75 (63, 88)
Severity I/II	189 (19%)	186 (19%)	169 (19%)	173 (20%)	285 (25%)	307 (27%)
III	532 (54%)	539 (54%)	445 (51%)	443 (52%)	633 (54%)	592 (52%)
IV	245 (25%)	244 (25%)	229 (26%)	204 (24%)	216 (19%)	220 (19%)
Unknown	21 (2%)	25 (3%)	30 (3%)	26 (3%)	18 (2%)	27 (2%)

Calculations subject to rounding errors

Numbers are median (25/75th centiles continuous data), *n* (%) for categorical. Rounding errors

*ICS* Inhaled corticosteroids, *yrs* years, *Pred* predicted

### Comparison of ICS Groups

When those patients taking ICS were divided between FP and other ICS groups, there were no significant differences in the baseline characteristics. There were 1981 patients in the FP group and 1719 in the other ICS group. Similarly, there were no significant differences when the groups were further subdivided into tiotropium or placebo pairs.

### Pneumonia Events

There were 854 patients with at least one pneumonia event (with 1121 events in total) (Supplementary Table 1). The total person-years in study was 17,721. Table 2 also summarises the numbers of pneumonia events in individual patients, i.e. 688 patients with one pneumonia event, 228 with two pneumonia events, etc. Predicted incidence rates are plotted in Supplementary Fig. 1, and the incidence rate ratios (IRRs) are shown in Table 3.

**Table 2:** Distribution of pneumonia events and incidence rates

Treatment	Events	Years in study	Incidence rate	Incident rate ratio (95% CI)	*p* value
(A)					
No ICS	383	6885	0.056		Reference
ICS	738	10,836	0.068	1.22 (1.08, 1.38)	0.012
(B)					
FP	437	5685	0.077	1.38 (1.20, 1.58)	<0.001
Other ICS	301	5151	0.058	1.05	0.52
No ICS	383	6885	0.056		Reference
(C)					
FP/Plac	220	2720	0.081	1.45 (1.19, 1.77)	<0.001
FP/tio	217	2964	0.073	1.31 (1.08, 1.60)	0.006
Other ICS/plac	153	2461	0.062	1.12 (0.90, 1.38)	0.29
Other ICS/tio	148	2690	0.055	0.99 (0.79, 1.23)	0.94
No ICS/plac	184	3317	0.055		Reference
No ICS/tio	199	3567	0.056	1.00 (0.82, 1.22)	0.95

*ICS* Inhaled corticosteroids, *FP* fluticasone propionate, *tio* tiotropium, *plac* placebo

**Table 3:** Incidence of COPD exacerbations by treatment group

Treatment	Exacerbations	Incidence	Incident rate ratio	*p* value
(A)				
No ICS	4256	0.62	0.62	Reference
ICS	9618	0.88	1.45 (1.36, 1.55)	<0.001
(B)				
FP	5292	0.93	1.52 (1.41, 1.64)	<0.001
Other ICS	4326	0.84	1.38 (1.27, 1.43)	<0.001
No ICS	4256	0.62		Reference
(C)				
FP/Plac	2798	1.03	1.57 (1.41, 1.75)	<0.001
FP/tio	2494	0.84	1.27 (1.14, 1.41)	<0.001
Other ICS/plac	2178	0.88	1.37 (1.22, 1.68)	<0.001
Other ICS/tio	2148	0.80	1.20 (1.07, 1.33)	0.001
No ICS/plac	2207	0.67		Reference
No ICS/tio	2049	0.57	0.86 (0.77, 0.95)	0.005

Calculations subject to rounding errors

Incidence rate ratios (IRRs) estimated from negative binomial regression

*ICS* Inhaled corticosteroids, *FP* fluticasone propionate, *tio* tiotropium, *plac* placebo

### Any ICS versus None

**Fig. 1:**
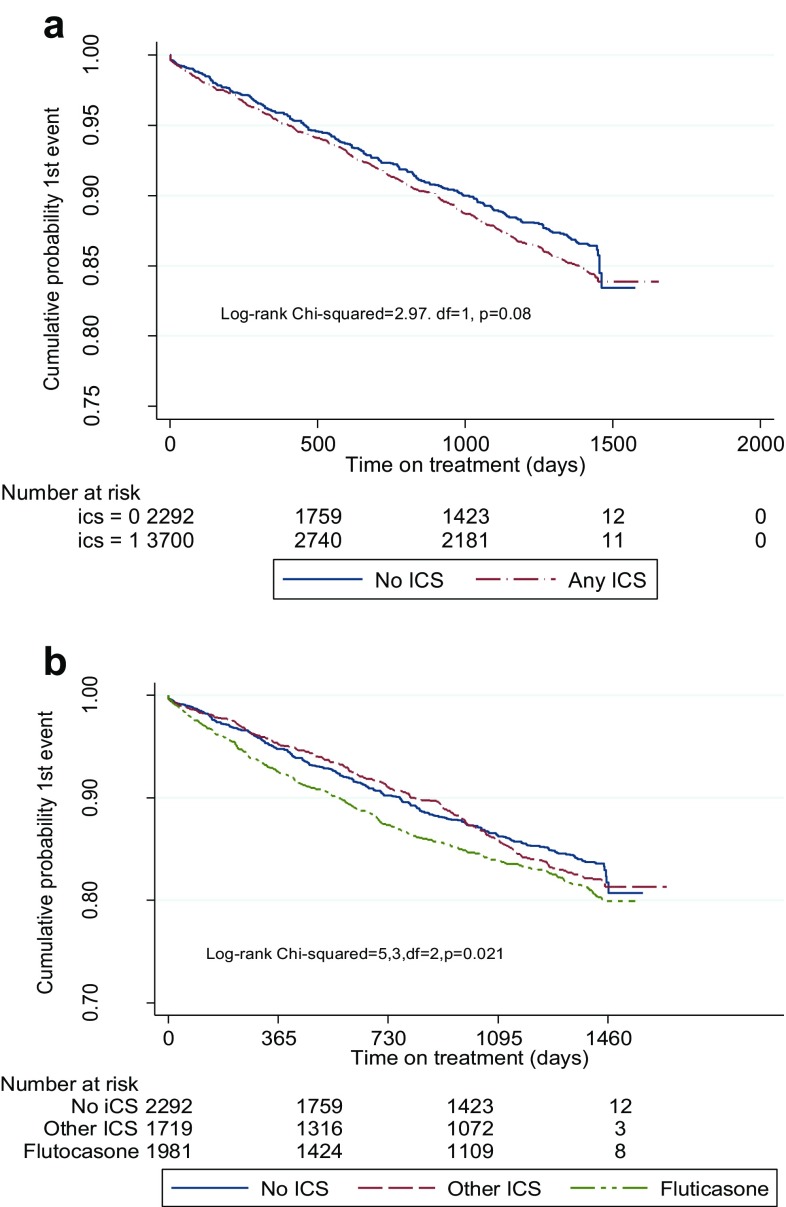
**a** Time-to-1st pneumonia event: ICS versus no ICS. **b** Time-to-first pneumonia event: Fluticasone versus other ICS vs no ICS

The distribution of pneumonia events by treatment group is given in Table 2a along with person-years exposure. The incidence rates of pneumonia was significantly higher in ICS patients compared with those with none (0.068 vs. 0.056, respectively; *p* = 0.012) (Fig. 1a). A similar trend was observed in the time-to-first event.

### FP Versus Other ICS versus None

The distribution of pneumonia events by treatment group is given in Table 2b. The incidence rates were highest for the FP patients (0.077); lowest was for the no ICS group (0.058). An overall pair-wise comparison found a significant difference between FP (higher incidence) and the other ICS reference group (*p* < 0.001). An attenuated rate of pneumonia, irrespective of ICS, was mainly found in the tiotropium subgroup (Table 2c).

### Time-to-1st Pneumonia Event

There was a significant difference between the time-to-first pneumonia event and treatment group with a shorter duration to first event in the FP group (*p* = 0.021) (Fig. 1b).

### COPD Exacerbations

There were 4050 patients who had at least one COPD exacerbation (1942 had none). Supplementary Table 2 summarises the number of subjects who had COPD exacerbations and their frequencies. The overall incidence of COPD exacerbations was 0.81 per person-years treatment.

### ICS versus None

Table 3a presents exacerbation rate by treatment group. There were significant differences (more COPD exacerbations) in the ICS groups compared to the group of patients not taking ICS (0.88 vs. 0.62; *p* < 0.001).

### FP Versus Other ICS Versus None

There was a significant (>10%) higher incidence of COPD exacerbation in FP group compared to those on other ICS (*p* = 0.013) (Table 3b) (Supplementary Fig. 2).

### Time-to-1st COPD Exacerbations

**Fig. 2:**
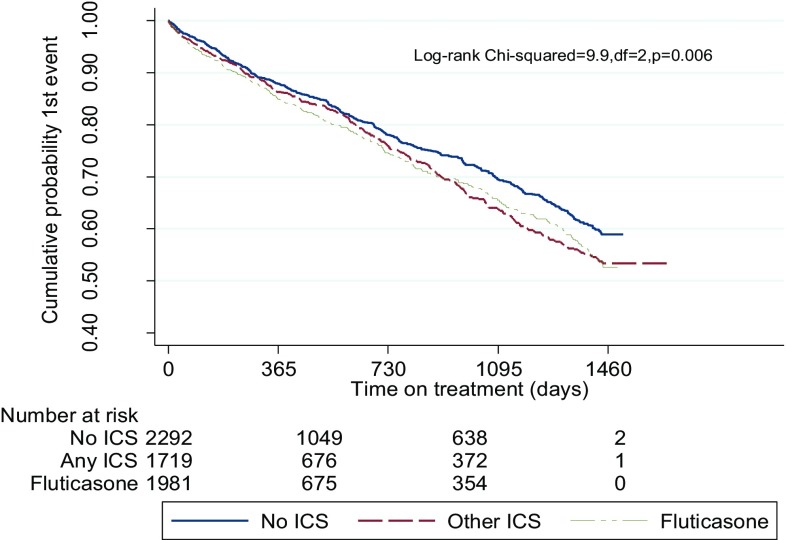
Time-to-1st COPD exacerbation: Fluticasone versus other ICS versus no ICS

The hazard ratio was lowest in the non-ICS group and highest in the FP/placebo group (Table 4). There was no significant difference between the FP and the other ICS treatments. The Kaplan–Meier curves demonstrate the separation between the two ICS (FP and other ICS) groups and patients on no ICS which is apparent from 2 years and persists to the end of study (Fig. 2).

**Table 4:** Cox regression for time-to-1st COPD exacerbation

Treatment group	HR (95% CI)	*p* value
(A)		
Any ICS	1.37 (1.29, 1.47)	0.001
None	Reference	
(B)		
Fluticasone	1.12 (1.04, 1.21)	0.003
Other ICS	Reference	
No ICS	0.77 (0.71, 0.83)	<0.001
(C)		
Fluticasone		
Placebo	1.16 (1.04, 1.29)	0.005
Tiotropium	0.96 (0.86, 1.07)	0.49
Other ICS		
Placebo	Reference	
Tiotropium	0.88 (0.78, 0.98)	0.031
No ICS		
Placebo	0.77 (0.69, 0.86)	<0.001
Tiotropium	0.67 (0.60, 0.75)	<0.001

Calculations subject to rounding errors

*ICS* inhaled corticosteroids, *HR* hazard ratio, *CI* confidence interval

## Discussion

Our analysis of this large 4-year prospective randomised study has established that ICS use at baseline is associated with an increase in both pneumonia (>20%) and COPD exacerbations (45%) compared to those not on ICS. The ITT principle was used in these analyses because of the difficulties in ascertaining the use and adherence to ICS therapy over a duration of the 4 years of study. ITT, although not ideal, was considered the safer analytic method in this secondary endpoint dataset. To support our assumptions, we conducted a number of sensitivity analyses without evidence of any interactions (see supplementary methods). As anticipated, the two groups were not matched at baseline in severity, with a quarter of ICS subjects in GOLD stage IV vs 19% in the no ICS group. Similarly FEV1% predicted in ICS group was 38% compared to 41% in the no ICS group. Thus the uneven distribution of baseline characteristics make it impossible to determine whether the differences in outcome are due to the adverse effects of treatment or the greater disease severity in those receiving the more intensive treatment.

In contrast, the 2 subgroups of patients receiving ICS that is, FP or other ICS, were very well matched in all the baseline characteristics. Thus, it is legitimate to compare the two treatment regimes on an intention-to-treat basis. In this study, we found a significant increase in the rate and number of cases of pneumonia and a significant increase in the number, but not the time-to-first event of COPD exacerbations. This suggests that any effect was disproportionately exhibited in those with frequent exacerbations. As stated earlier, UPLIFT differs from other therapeutic trials in COPD in that concomitant medications were permitted throughout [23]. Thus, the increase of nearly a third in the episodes of pneumonia seen in our analysis poinst to an adverse effect of FP therapy rather than other factors such ICS withdrawal as seen in other studies. Indeed, when the frequency of pneumonia in the no ICS group is compared to the other ICS, rates are similar. Thus, almost all of the excess of pneumonia episodes due ICS treatment can be ascribed to FP (Table 2).

Episodes of COPD exacerbations were also increased in the FP group. However, the time-to-first exacerbation was initially similar across the 3 groups, before diverging with clear increase for both the ICS groups (Fig. 2). Given that there was less severe disease in the no ICS group, this suggests that time-to-first event may be a less good marker of outcome than frequency of exacerbation. We suggest that ICS, and particularly FP as stated above, may adversely affect those prone to recurrent exacerbations of COPD: Contrary to many guideline recommendations [1, 25].

Our findings confirm the observations of several randomised trials of varying durations and doses of FP. However, unlike the 2-year INSPIRE and 3-year TORCH studies where high HRs of pneumonia were reported [10, 11], our analysis demonstrates a significant but more modest effect. It is also much lower than other shorter duration randomised trials and of studies with lower FP doses [12, 13]. We suggest that this may be due to the longer observation period of 4 years in UPLIFT. Retrospective database and case–control analyses of longer durations have also reported higher rates of pneumonia [18, 19].

Our observations are similar to the retrospective case–control PATHOS study which found lower rates of COPD exacerbations and pneumonia in patients treated with budesonide/formoterol compared to FP/salmeterol [19, 26]. In a study of dual bronchodilators (LAMA/LABA combination) versus FP/salmeterol, the latter also resulted in significant increases in pneumonia and COPD exacerbations in moderate-to-severe patients [27]. The superiority of dual bronchodilation over ICS/LABA has also been confirmed in the recent FLAME study [6].

Fluticasone differs from other ICS, such as beclomethasone and budesonide, in the presence of a fluorine moiety. This drags the electrons across the molecule altering not only potency but also lipid solubility [28, 29]. In vivo this is characterised by an alteration in the volume of distribution, fluticasone dwelling for longer in the lipid membranes. The consequence of this is a slower clearance from the lungs and other tissues. This will clearly have effects on lung immunity and epithelial barrier function [30]; thus, dampening down inflammatory responses. Alternatively, potent steroids may increase the risk of potential aspiration events. That fluticasone particularly prone to these adverse effects is confirmed by recent reports of increased pneumonia with fluticasone furoate in COPD [31, 32]. Our analysis does not exonerate other ICS from adverse respiratory outcomes; it merely suggests that these are more prevalent with fluticasone. This is supported by a recent study suggesting that withdrawal of ICS decreases the risk of pneumonia and that this is particularly marked with FP [33]. In ICS withdrawal study, WISDOM, a short-term increase in adverse respiratory events was observed on gradual steroid withdrawal from triple inhaled therapy [34]. In contrast, in the FLAME study, abrupt withdrawal of ICS did not increase exacerbation rates [6]. Asthma COPD overlap syndrome (ACOS) [35] may explain some of the anomalies seen in these studies.

A clear observation from this analysis is that long-acting bronchodilator therapy in the form of tiotropium ameliorates some of the adverse effects of ICS treatment with the incidence rates returning towards those patients taking other ICS. We suggest that the use of ICS without LAMA may expose the patient to additional risk of respiratory adverse events.

We conducted the analyses on the classic basis of intention to treat despite this our observations have strengths and weaknesses. This large study population was observed over a 4-year period in patients with a range of airflow limitation and COPD severity. Co-existing treatment with ICS was uninfluenced either at study entry or during the course of the study. This avoids a major source of bias. A downside of this analysis is that we have not corrected for withdrawal or changing of ICS during the course of the study. In this regard, we are conducting the analysis on the basis of ‘intention to treat’. A further limitation of this study is the reliance on secondary endpoints which were less tightly defined. Thus, exacerbations of COPD were defined as an increase in new onset of at least or more symptoms for >3 days which required additional treatment with antibiotics and/or corticosteroids. It is likely therefore that some cases of pneumonia were included in this loose definition. Pneumonia was only defined as an investigator-reported adverse event not necessarily requiring confirmation by chest X-ray. In contrast to several large database studies with similar findings, the prospective nature and rigid diagnostic criteria for entry into UPLIFT ensures that our analysis has been performed on a clearly defined COPD population.

## Conclusion

In conclusion, our analyses have shown that the use of FP in patients with COPD is associated with a significant risk of pneumonia and increase in COPD exacerbations compared to patients on other ICS or those not taking any ICS. Although our observations are a secondary analysis of the large UPLIFT study, the magnitude of this intra-class difference needs to be put into context when choosing optimal therapy for COPD patients. Our results add further weight to the body of evidence cautioning against the use of fluticasone in this disease.

## Electronic supplementary material

Below is the link to the electronic supplementary material.

Supplementary material 1 (PPTX 56 KB)

Supplementary material 2 (DOCX 20 KB)

Supplementary material 3 (DOCX 17 KB)

Supplementary material 4 (DOCX 17 KB)
