# Supplementary figures and images for: Inhaled Corticosteroid use and the Risk of Pneumonia and COPD Exacerbations in the UPLIFT Study
Source: Lung. 2017 Mar 3;195(3):281–8. doi: 10.1007/s00408-017-9990-8 (PMC5437199; doi:10.1007/s00408-017-9990-8)

## Slide 1
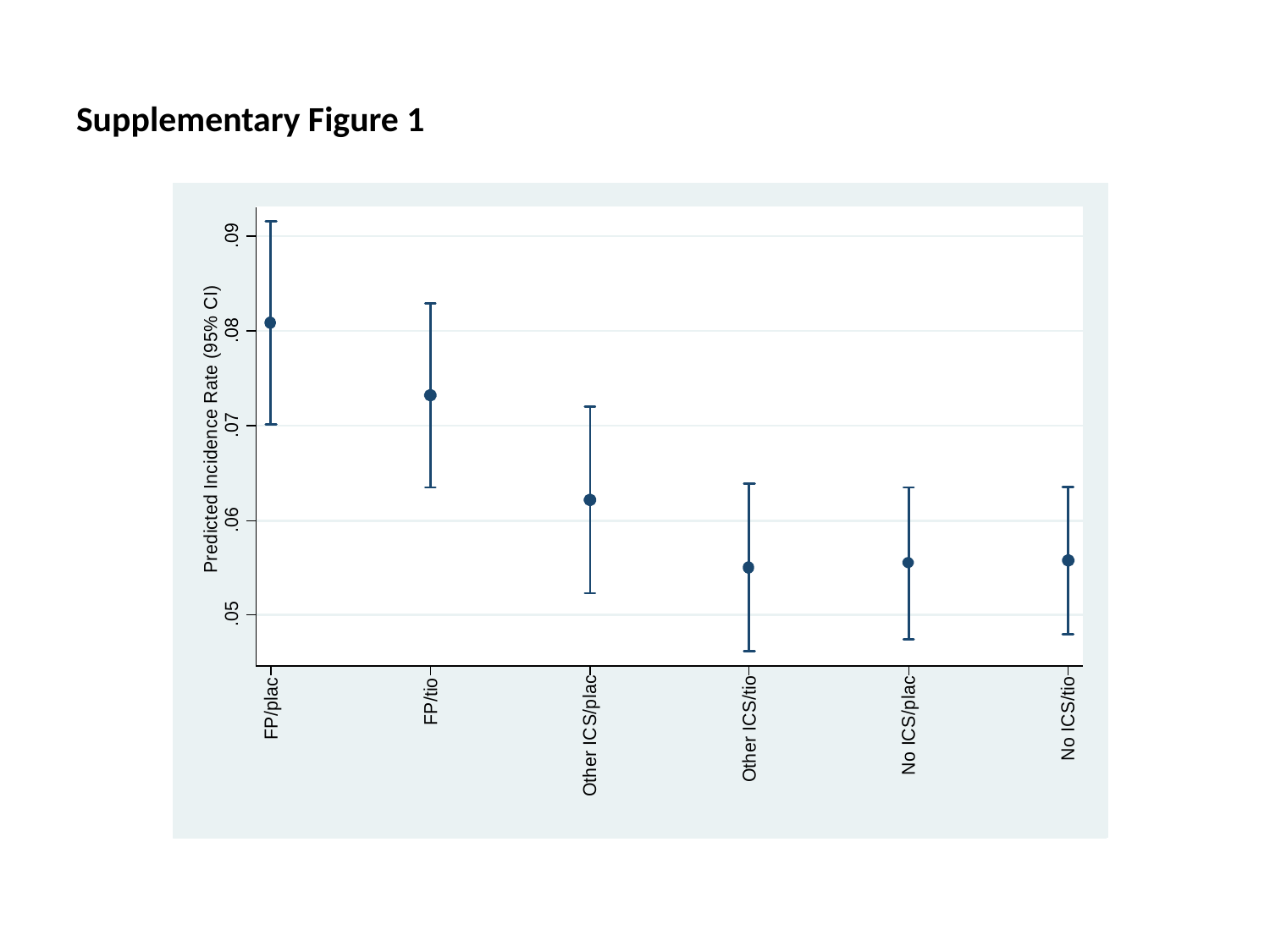

# Supplementary Figure 1

## Slide 2
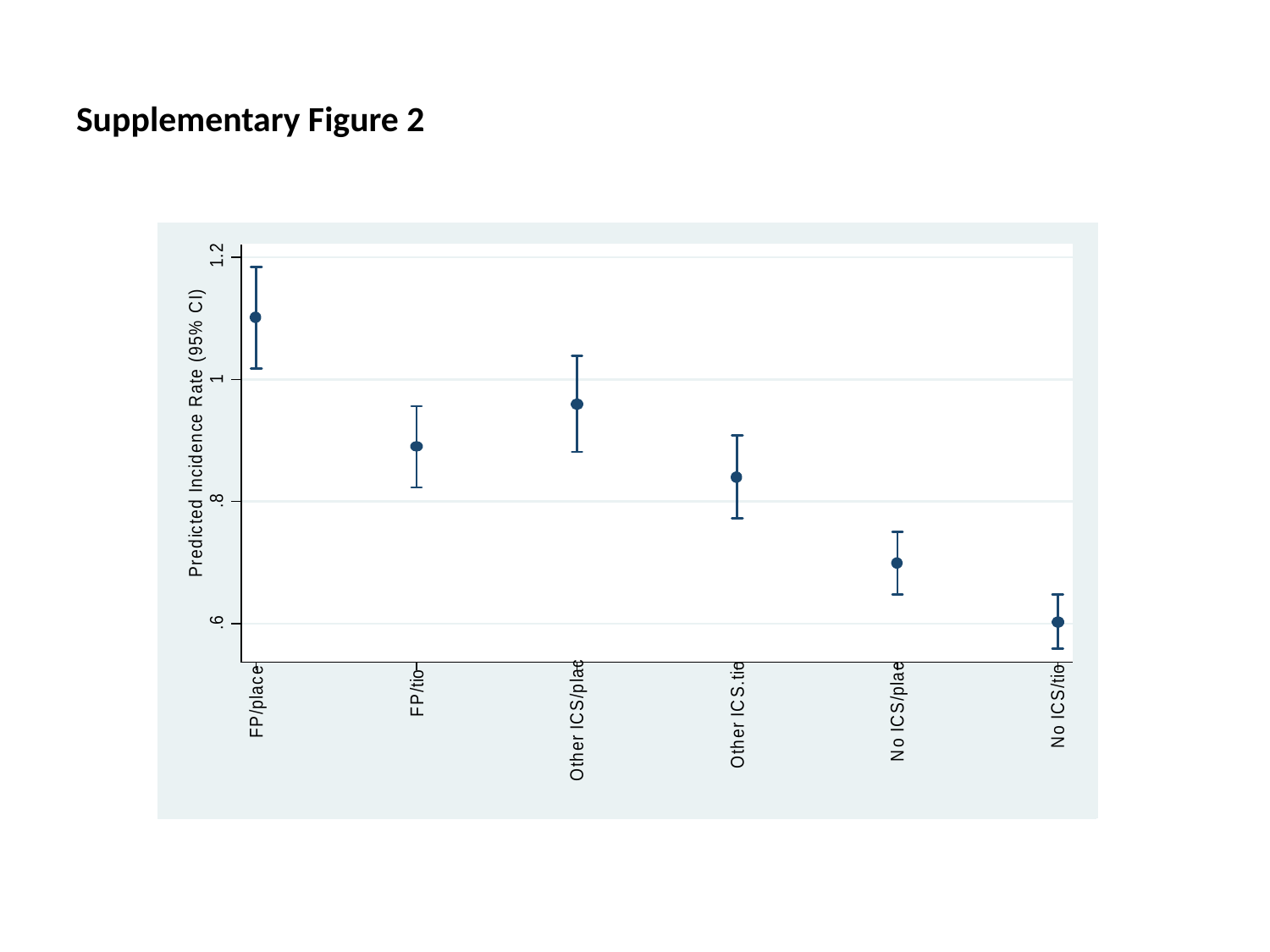

# Supplementary Figure 2

Supplement: Supplementary file 1 — Supplementary material 1 (PPTX 56 KB) [file 408_2017_9990_MOESM1_ESM.pptx]
