# Supplementary material for: Inhaled Corticosteroid use and the Risk of Pneumonia and COPD Exacerbations in the UPLIFT Study
Source: Lung. 2017 Mar 3;195(3):281–8. doi: 10.1007/s00408-017-9990-8 (PMC5437199; doi:10.1007/s00408-017-9990-8)
Supplement: Supplementary file 2 — Supplementary material 2 (DOCX 20 KB) [file 408_2017_9990_MOESM2_ESM.docx]

**Inhaled corticosteroid use and the risk of pneumonia and COPD exacerbations in the UPLIFT study**

**Jaymin Bhagwanji Morjaria^1, 2^ MBBS, MD; Alan Rigby^1^ CStat; Alyn Hugh Morice^1^ MBBChir MD.**

1. Centre for Cardiovascular & Metabolic Research, Hull York Medical School, University of Hull, Castle Hill Hospital, Castle Road, Cottingham HU6 5JQ.
2. Dept of Respiratory Medicine, Royal Brompton & Harefield NHS Trust, Harefield Hospital, Hill End road, Harefield UB9 6JH.

Corresponding Author and Address:

Prof Alyn H Morice

Centre for Cardiovascular & Metabolic Research

Hull York Medical School

University of Hull

Castle Hill Hospital

Castle Road

Cottingham HU6 5JQ.

Tel: 01492624067

Fax: 01482624068

Email: a.h.morice@hull.ac.uk

**Contributions -** JBM and AHM were involved in the concept, interpretation of the data, writing of the manuscript. ASR was involved in the statistical analyses and the writing of the manuscript.

**Sources of Support**: None

**Running Title**: ICS, exacerbations and pneumonia in UPLIFT

**Key Words**: Fluticasone, inhaled corticosteroids, tiotropium, UPLIFT, pneumonia, COPD

**Word Count:** Abstract - 250; Body of manuscript - 2681 (excl Abstract, legends, references)

**Online Supplement:** This article has an online data supplement.

**Supplementary Methods**

**Statistical Analyses**

‘Overdispersion’ in Poisson data occurs when the variance is much greater than the mean; this is analysed using negative binomial regression ([1](#_ENREF_1))**.** There was no evidence for overdispersion for pneumonia events (variance=0.33, mean=0.18), however the frequency of COPD exacerbations was overdispersed (variance=8.76, mean=2.31). Predicted event rates from either Poisson regression (pneumonia) or negative binomial regression (COPD exacerbations) are summarized graphically with 95% confidence intervals (CIs). Treatment groups were compared statistically using incident rate ratios (IRRs), and pairwise comparisons made where necessary.

For the Cox regression analysis, the proportionality of hazards was confirmed by a test based on Schoenfeld residuals ([2](#_ENREF_2)). The Cox regression model was not overfitted ([3](#_ENREF_3)). Goodness-of-fit was measured by Harrell’s concordance (C) statistic, but noting that it is overoptimistic for censored survival data ([4](#_ENREF_4)). Harrell’s C is defined as the probability that predictions and outcomes are concordant.

*Sensitivity Analyses*

We chose to use ITT analyses for reasons of the greater accuracy of the baseline data as commented on in the first paragraph of the discussion. We recognise that this may be criticised because of the lack of adjustment for treatment change during the trial period. To examine whether there was significant interactions between treatment groups we conducted an analyses of baseline variables; there was no significant interactions between the following variables: ICS and gender (p=0.88); the reported use of fluticasone and gender (p=0.70). We examined interactions between all 6 cohorts and gender, and again found no significant interaction (p=0.88). Next we examined the interaction of smoking and treatment. There was no significant interaction with ICS (p=0.79), fluticasone (p=0.53) and the entire cohort (p=0.8). Finally we examined the interaction of treatment with morbidity, there was no significant interaction with ICS use (p=0.52), fluticasone (p=0.20) and the entire treatment cohort (p=0.34). We suggest that the lack of interaction above indicates the validity of our assumption of using ITT analyses.

**References**

1. Lawless JF. Negative binomial regression and mixed Poisson regression. *The Canadian Journal of Statistics* 1987; 15: 209-215.

2. Schoenfeld D. Partial residual estimation for the proportional hazards regression. *Biometrika* 1982; 69: 239-241.

3. Peduzzi P, Concato J, Feinstein AR, Holford TR. Importance of events per independent variable in proportional hazards regression analysis. II. Accuracy and precision of regression estimates. *J Clin Epidemiol* 1995; 48: 1503-1510.

4. Uno H, Cai T, Pencina MJ, D'Agostino RB, Wei LJ. On the C-statistics for evaluating overall adequacy of risk prediction procedures with censored survival data. *Stat Med* 2011; 30: 1105-1117.
