# Supplementary material for: Inhaled Corticosteroid use and the Risk of Pneumonia and COPD Exacerbations in the UPLIFT Study
Source: Lung. 2017 Mar 3;195(3):281–8. doi: 10.1007/s00408-017-9990-8 (PMC5437199; doi:10.1007/s00408-017-9990-8)
Supplement: Supplementary file 3 — Supplementary material 3 (DOCX 17 KB) [file 408_2017_9990_MOESM3_ESM.docx]

**Supplementary Table 1. Distribution of pneumonia events by treatment group.**

Treatment group

Pneumonia Fluticasone Other ICS No ICS Total Patients

Events Pl Tio Pl Tio Pl Tio

987 994 873 846 1146 1146 5992

------------------------------------------------------------------------------------------------

0 844 831 747 730 997 989 5138 Total Events

------------------------------------------------------------------------------------------------

1 109 131 103 97 122 126 688 688

2 20 18 19 12 22 23 114 228

3 5 9 4 5 2 5 30 90

4 1 3 1 3 3 11 44

5 5 1 6 30

6 1 1 6

7 1 1 7

8 1 1 2 16

12 1 1 12

------------------------------------------------------------------------------------------------

Events 220 217 153 148 184 199 1121

------------------------------------------------------------------------------------------------

Abbreviations: Pl – placebo; ICS – inhaled corticosteroids; Tio – tiotropium.
