# Supplementary material for: Inhaled Corticosteroid use and the Risk of Pneumonia and COPD Exacerbations in the UPLIFT Study
Source: Lung. 2017 Mar 3;195(3):281–8. doi: 10.1007/s00408-017-9990-8 (PMC5437199; doi:10.1007/s00408-017-9990-8)
Supplement: Supplementary file 4 — Supplementary material 4 (DOCX 17 KB) [file 408_2017_9990_MOESM4_ESM.docx]

**Supplementary Table 2. Distribution of COPD events by treatment group.**

Treatment group

COPD Fluticasone Other ICS No ICS Patients

Exacerbations Pl Tio Pl Tio Pl Tio

987 994 873 846 1146 1146 5992

--------------------------------------------------------------------------------------------------------------------------------

0 262 288 260 250 435 447 1942 Exacerbations

--------------------------------------------------------------------------------------------------------------------------------

1 201 195 199 173 243 249 1260 1260

2 144 148 100 112 149 176 829 1658

3 52 97 89 81 94 88 541 1623

4 64 75 65 72 80 52 408 1632

≥5 244 191 160 158 145 134 1012 7701

---------------------------------------------------------------------------------------------------------------------------------

Exacerbations 2798 2494 2178 2148 2207 2049 13874

---------------------------------------------------------------------------------------------------------------------------------

Abbreviations: Pl – placebo; ICS – inhaled corticosteroids; Tio – tiotropium.
